# A Novel Bi-Directional and Bi-Temporal Delivery System for Enhancing Intrasynovial Tendon Repair

**DOI:** 10.53941/mi.2024.100001

**Published:** 2024-10-18

**Authors:** Yidan Chen, Seth Kinoshita, Emily Yan, Min Hao, Hua Shen, Richard Gelberman, Stavros Thomopoulos, Younan Xia

**Affiliations:** 1 School of Materials Science and Engineering, Georgia Institute of Technology, Atlanta, GA 30332, USA; 2 School of Chemistry and Biochemistry, Georgia Institute of Technology, Atlanta, GA 30332, USA; 3 The Wallace H. Coulter Department of Biomedical Engineering, Georgia Institute of Technology and Emory University, Atlanta, GA 30332, USA; 4 Department of Orthopedic Surgery, Washington University School of Medicine, St. Louis, MO 63110, USA; 5 Department of Orthopedic Surgery, Department of Biomedical Engineering, Columbia University, New York, NY 10032, USA

**Keywords:** flexor tendon repair, sodium hyaluronate, drug delivery, controlled release, Transwell cell culture

## Abstract

Flexor tendon injuries are common and often require surgical repair and prolonged rehabilitation. Successful clinical outcomes depend on the concurrent suppression of adhesions (caused by inflammation) at the tendon surface and promotion of matrix synthesis inside the tendon. Herein, we report a bi-directional and bi-temporal drug delivery system designed to target both the initial inflammatory phase and the subsequent proliferative and remodeling phases of healing to improve outcomes after flexor tendon repair. The system features a multi-layered design with anti-adhesion and pro-matrix factors encapsulated in separate layers of hyaluronate films crosslinked to different degrees to control their direction and rate of release. After validating drug delivery under controlled release, cell culture experiments involving tendon fibroblasts and a Transwell system are conducted to demonstrate the system’s efficacy in modulating local cellular responses. The promising results from this study lay the groundwork for moving this system toward in vivo testing and clinical translation.

## Graphical Abstract

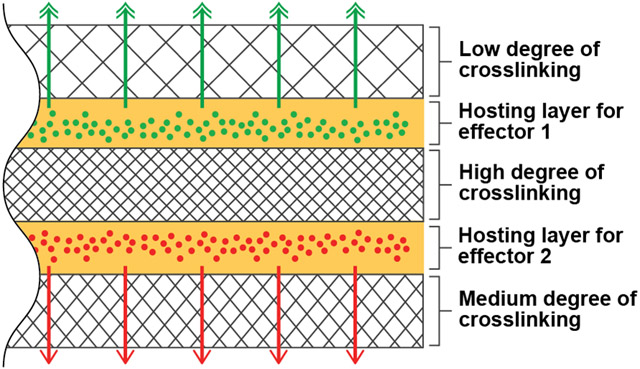

## 1. Introduction

The flexor tendons of the hand facilitate movements necessary for daily activities such as gripping and manipulating objects. Intrasynovial flexor tendon injuries, including lacerations and ruptures, are common and they result in significant pain and disability. Treatment of these injuries typically requires surgical repair and prolonged rehabilitation to regain function. Unfortunately, current approaches often fail to return full function to the hand due to poor healing that leads to gapping or repair-site-failure, and adhesion formation that limits motion. To improve the outcome of the repair of an intrasynovial flexor tendon injury, it is necessary to concurrently suppress the adhesion between the tendon and the surrounding sheath while accelerating the regeneration of the tendon at the repair site [1].

Recent research efforts have focused on applying stem cells, growth factors, and/or small molecule drugs to the tendon injury site following surgical repair to improve the outcome [2-5]. However, most delivery systems are designed to release a single factor at the target site, making them inadequate for flexor tendon repair, which requires simultaneous prevention of adhesion at the tendon surface and promotion of matrix synthesis inside the tendon. Herein, we report a novel bi-directional and bi-temporal controlled release system that can be wrapped around the intrasynovial tendon repair site to address this need. Specifically, this system delivers an anti-adhesion factor toward the tendon sheath during the early inflammatory period and a pro-matrix factor toward the tendon repair site in the subsequent proliferative and remodeling phases, effectively addressing both requirements for successful healing.

**Figure 1.**
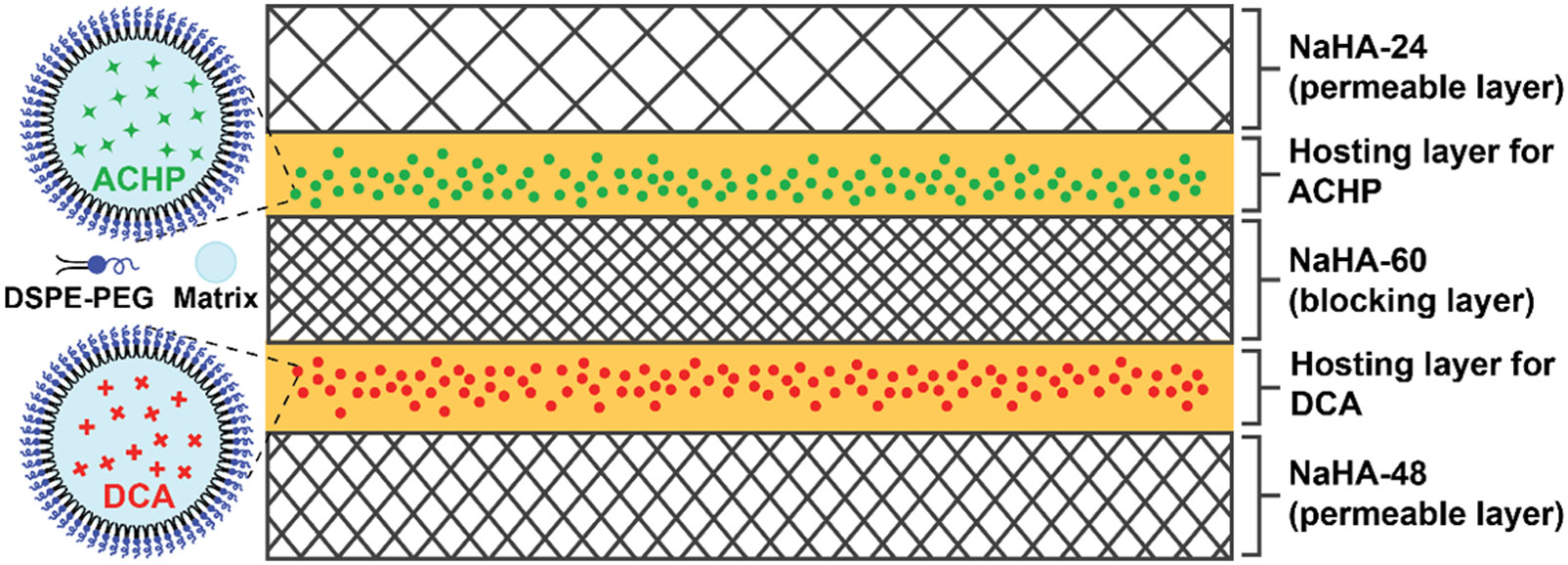
Schematic of the bi-directional and bi-temporal release system, which adopts a multi-layered structure, with two hosting layers containing fatty acid nanoparticles pre-loaded with the small molecule drugs, ACHP and DCA, respectively, and stabilized by DSPE-PEG surfactant. The hosting layers are comprised of a physically-crosslinked PVA-NaHA matrix. The two hosting layers are separated by a blocking layer made of NaHA-60. Two permeable layers, made of NaHA-24 and NaHA-48, respectively, help control the release kinetics of the ACHP- and DCA-loaded nanoparticles. NaHA-24, NaHA-48, and NaHA-60 correspond to NaHA films crosslinked for 24, 48, and 60 h in the presence of (1-ethyl-3-(3-dimethylaminopropyl) carbodiimide, respectively.

Our controlled release system is based on sodium hyaluronate (NaHA), a major lubricating component of the synovial fluid that has been extensively used in flexor tendon repairs [6,7]. Prior studies suggested that NaHA can prevent adhesion formation and reduce gliding resistance both in vitro and in vivo [8,9]. Therefore, NaHA is considered an excellent carrier material for delivering biological effectors in intrasynovial tendon repair. However, the half-life of NaHA in the physiological environment is very short (several hours to 1.5 days [10]) due to the presence of enzymes, such as hyaluronidase (HAase), capable of rapidly breaking down NaHA-based carriers once implanted. To increase the tissue residence time of a NaHA-based delivery system while enhancing its mechanical properties, here we use (1-ethyl-3-(3-dimethylaminopropyl) carbodiimide (EDC) to help crosslink the NaHA matrix into a network. By controlling the duration of crosslinking, we obtain NaHA films with different degrees of crosslinking under a condition compatible with the physiological system. The crosslinked NaHA films are fully characterized, and their drug delivery capabilities are examined, including the degradation rates and cumulative drug release profiles. To achieve bi-directional and bi-temporal release, the delivery system is designed to adopt a multi-layered structure, in which the payloads are encapsulated in two separate hosting layers between NaHA films crosslinked to different degrees (Figure 1). While the two sides of the system are capped by NaHA films (i.e., the permeable layers) crosslinked to different degrees to achieve bi-temporal release, a middle film made of the most heavily crosslinked NaHA serves as a blocking layer to prevent the inter-diffusion between the two drug-loaded layers, realizing bi-directional delivery. We explore two small molecule drugs, the IKKβ inhibitor 2-amino-6-(2-(cyclopropylmethoxy)-6-hydroxyphenyl)-4-(piperidin-4-yl)nicotinonitrile hydrochloride (ACHP hydrochloride, C_21_H_25_ClN_4_O_2_) (to control inflammation) and sodium dichloroacetate (DCA) (to control metabolism), in this study. To avert a potential detrimental burst release effect, the small molecule drugs are pre-loaded in nanoparticles made of natural fatty acids and then encapsulated in a hosting layer comprised of physically-crosslinked poly(vinyl alcohol) (PVA) and NaHA. The bi-directional and bi-temporal release is visualized and validated by replacing the small molecule drugs with fluorescent model compounds. A series of tendon fibroblast Transwell culture experiments are conducted to demonstrate the efficacy of the bi-directional and bi-temporal delivery system in modulating cellular responses. By establishing the biological effectiveness of the bi-directional delivery of ACHP and DCA in vitro, the current study poses a strong foundation for exploring the opportunity to concurrently prevent adhesion formation through IKKβ inhibition and enhance tendon strength through modulation of metabolism.

## 2. Experimental Section

### 2.1. Chemicals and Materials

Lauric acid (>98%), stearic acid (95%), ethanol (EtOH, HPLC grade), PVA (Mw ≈ 89,000–98,000), thiazolyl blue tetrazolium bromide (MTT), dimethyl sulfoxide (DMSO), rhodamine B (RhB), indocyanine green (ICG), DCA, hyaluronidase (from bovine testes, 400–1000 units/mg solid), and glass microcapillary tube (Ace Glass) were all obtained from Sigma-Aldrich (St. Louis, MO, USA). Fetal bovine serum (FBS), EDC hydrochloride, Dulbecco’s modified Eagle medium (DMEM) with a high glucose content (4.5 g/L), DMEM with a low glucose content (1 g/L) and sodium pyruvate (110 mg/L), antibiotic-antimycotic, phosphate-buffered saline (PBS, pH = 7.4), calcein-AM, ethidium homodimer-1 (EthD-1), TRIzol reagent, and PVC tubes were ordered from Thermo Fisher (Waltham, MA, USA). ACHP hydrochloride was obtained from MedChemExpress (Monmouth Junction, NJ, USA); 1,2-distearoyl-sn-glycero-3-phosphoethanolamine-N-[methoxy (polyethylene glycol)-5000] (Mw ≈ 5000, DSPE-PEG 5000) was purchased from Laysan Bio Inc (Arab, AL, USA); NaHA (Mw ≈ 200 k) was ordered from Lifecore Biomedical (Chaska, MN, USA); syringe needles (18 G and 26 G) were ordered from BD (Franklin Lakes, NJ, USA); and epoxy adhesive was obtained from Devcon. All chemicals and materials were used as received. The deionized (DI) water used in all experiments was obtained by filtering through a Millipore ultrapure water system (Billerica, MA, USA).

### 2.2. Fabrication of Fatty Acid Nanoparticles and Encapsulation of Payloads

Fatty acid nanoparticles were prepared through hydrodynamic focusing in a homemade fluidic device by following a published protocol [11]. Briefly, the fluidic device was fabricated by inserting a 26 G needle into a PVC tube (inner diameter: 1/16 inch) and connecting it with a microcapillary tube (550 μm inner diameter), followed by fixing and sealing with epoxy adhesive. The focused phase, a solution of fatty acids, was prepared by dissolving lauric acid and stearic acid at a 4:1 mass ratio in EtOH with a total concentration of 6 mg/mL. The focusing phase was prepared by dissolving the surfactant DSPE-PEG in water at a concentration of 0.2 mg/mL. The fatty acid and surfactant solutions were introduced using two syringe pumps (KD100, KD Scientific, Holliston, MA, USA) with independently controlled flow rates. The flow rates of the focused phase and focusing phase were set to 14 and 700 μL/min, respectively. The encapsulation of the fluorescent molecules (RhB and ICG), DCA, and ACHP in the fatty acid nanoparticles was achieved by directly adding them into the fatty acid solution. For molecules that are scarcely soluble in EtOH, including ICG and ACHP, they were dissolved in DMSO at specific concentrations (10 mg/mL for ICG and 25 mg/mL for ACHP), followed by mixing with the fatty acid solution in ethanol at a 5:95 (*v/v*) ratio. For compounds highly soluble in EtOH (e.g., RhB and DCA), the powders were directly dissolved in the fatty acid solution in EtOH at a concentration of 20 mg/mL for RhB and 30 mg/mL for DCA at 50 °C, respectively. The nanoparticles were collected and kept in an ice bath, except for washing. The nanoparticles were washed with water containing DSPE-PEG (0.5 mg/mL) three times at 16,600 g rcf for 8 min using a centrifuge (5471C and 5810, Eppendorf, Hamburg, Germany) to remove the free drug molecules. The nanoparticles were finally resuspended in water containing DSPE-PEG (1 mg/mL) for further use.

### 2.3. Preparation and Characterization of the Crosslinked NaHA Films

The NaHA films were prepared through solution casting, followed by crosslinking in a 50 mM EDC solution in an EtOH/water mixture (8:2, *v/v*) for different periods of time. The polymer solution was prepared by dissolving NaHA in water at a concentration of 1 wt.% under stirring overnight on an orbital shaker (Apollo Digital Orbital Shaker, CLP) at 300 rpm and room temperature. In a typical process, 5 mL of 1 wt.% NaHA was cast in a glass petri dish (VWR, Radnor, PA, USA) with a diameter of 50 mm. The dish was placed in a fume hood with a laminar flow for 20 h to allow the solvent to evaporate. The dried NaHA film was peeled off and then cut into smaller pieces of the desired size (circles with a diameter of 1 inch). The crosslinking solution was prepared by dissolving EDC in the EtOH/water mixture and shaking at 300 rpm for 1 h to obtain a homogeneous mixture. To achieve varying degrees of crosslinking, the NaHA films were immersed in the crosslinking solution for 24, 48, and 60 h at room temperature. The resultant films are denoted NaHA-24, NaHA-48, and NaHA-60, respectively, according to the duration of crosslinking. After crosslinking, the films were washed with the EtOH/water mixture for 3 min, followed by rinsing with 50% EtOH and H_2_O and finally drying at room temperature in a fume hood with laminar flow. Infrared (IR) spectra of the non-crosslinked NaHA, NaHA-24, NaHA-48, and NaHA-60 films were recorded using a Fourier Transform Infrared (FT-IR) Spectrometer (Shimadzu IRAffinity-1, Shimadzu, Kyoto, Kyoto, Japan) under attenuated total reflection mode.

### 2.4. Degradation of the Crosslinked NaHA Films

The degradation of the NaHA films with different degrees of crosslinking was evaluated in vitro by immersing the NaHA-24, NaHA-48, and NaHA-60 films in 1 mL of PBS containing 20 U HAase and incubating at 37 °C [12]. The medium was changed every day. For each type of film, multiple (N = 3) pieces were used for mass loss measurement at each time point. Briefly, the medium was removed, and the films were then freeze-dried for 5 h to remove any remaining liquid before being weighed on a balance. The percentage mass remaining for each group was calculated by normalizing the value against the starting mass of the respective group.

### 2.5. Monitoring the Release of Fatty Acid Nanoparticles from the Crosslinked NaHA Films

Fluorescently-labeled fatty acid nanoparticles were prepared by encapsulating RhB and ICG using the antisolvent precipitation method described above. A mixture of PVA (7.5 wt.%) and NaHA (0.5 wt.%) was prepared by mixing 15 wt.% PVA and 1 wt.% NaHA at 1:1 *(v/v)* ratio and vortexing for 1 min on a Vortex-Genie 2 vortex mixer (Scientific Industries, NY, USA) to obtain a homogeneous solution. This mixture was used both as the hosting layer for the drug-loaded fatty acid nanoparticles and as a glue during the layer-by-layer assembly process. The fluorescently labeled nanoparticles were loaded between NaHA films with different degrees of crosslinking. Release from NaHA-24 and NaHA-48 were monitored using the structure in Figure S1A, and that from NaHA-60 was monitored using the structure in Figure S1B. After the assembling process, the structure went through three freeze-thawing cycles for the PVA in the PVA-NaHA mixture to form hydrogen bonds. One freeze-thawing cycle involved freezing at −80 °C for 3 h and thawing at 4 °C for 1 h. The structure was subsequently incubated in 150 μL of PBS, or PBS containing 10 U or 20 U HAase at 37 °C [12,13]. For each sample, the incubation solution was removed every 24 h and 150 μL of fresh solution of the corresponding type was supplemented back. The amount of nanoparticles released was correlated with the reduction of fluorescence intensity within the system. Fluorescence images were recorded using a confocal fluorescence microscope (Zeiss LSM 900, Zeiss, Oberkochen, Germany) every 24 h for the first 5 days and every 48 h for the remaining period. The beam intensity, exposure time, and digital gain were fixed for each sample. At each time point, images were taken at 10 random spots on a single sample and the average fluorescence intensity of these spots was recorded. The release for each sample was normalized against the fluorescence intensity recorded at the beginning of the experiment (*t* = 0 h). Three technical replicates with the same degree of crosslinking and the same incubation condition were performed for each group (N = 3).

### 2.6. Fabrication of the Bi-Directional and Bi-Temporal Delivery System

We fabricated the delivery system by assembling the NaHA films in a layer-by-layer manner. In a typical process, a NaHA-48 film was placed at the bottom and then a thin layer of the PVA-NaHA mixture was applied by brush coating. Afterward, 50 μL of the suspension of DCA-loaded fatty acid nanoparticles containing a total dose of 1.483 mg (9.83 μmol) DCA was dropped on the top. The PVA-NaHA mixture was evenly applied to the bottom of a NaHA-60 film and the film was attached to the previous structure, with the PVA-NaHA side facing down. The same procedure was repeated for the top layers containing the ACHP-loaded nanoparticles: a 10 μL suspension of ACHP-loaded fatty acid nanoparticles containing a total dose of 22.3 μg (56 nmol) ACHP was sandwiched between the middle layer of NaHA-60 and the topmost layer of NaHA-24. After the assembly process, the delivery system went through three freeze-thawing cycles. The thickness of the dried construct was around 46.0 μm.

### 2.7. Visualization of the Bi-Directional Release

The bi-directional release from the multi-layered NaHA construct was visualized using fatty acid nanoparticles pre-loaded with fluorescent model compounds. Specifically, ICG and RhB were selected to represent ACHP and DCA, respectively. The construct was incubated in 200 μL of 20 U HAase-supplemented PBS solution at 37 °C in a 12-well plate for up to 6 days [12]. Z-stack confocal fluorescence micrographs were captured every day using a confocal fluorescence microscope (Zeiss LSM 900). Cross-sectional images were reconstructed from the z-stack ones using the Zen (blue) software from Zeiss.

### 2.8. Fabrication of a Unidirectional Delivery System for the Cell Culture Experiment

A simplified unidirectional delivery system was used in the in vitro experiment (Figure S2). The system was fabricated and attached to the Transwell insert in a similar manner to the bi-directional and bi-temporal delivery system, except that only two layers of NaHA films were involved. The system was attached to the bottom of a Transwell insert (6-well insert, 0.4 μm, PET translucent, cellQART, SABEU, Northeim, Germany) with the same PVA-NaHA mixture as described above, covering the Transwell membrane entirely from the bottom. The PVA-NaHA mixture was carefully applied to the peripheral region of the film only. Three freeze-thawing cycles were carried out to securely bond the delivery system to the Transwell membrane. For the DCA experiment, DCA-loaded fatty acid nanoparticles were hosted in a PVA-NaHA mixture layer sandwiched between a NaHA-48 film and a NaHA-60 film. The system was attached to the bottom of the Transwell insert with the NaHA-60 film facing the membrane. For the ACHP experiment, ACHP-loaded fatty acid nanoparticles were hosted in a PVA-NaHA mixture layer sandwiched between a NaHA-60 film and a NaHA-24 film. The system was attached to the bottom of the Transwell insert with the NaHA-24 film facing the membrane.

### 2.9. Isolation, Culture, and Seeding of Tendon Fibroblasts

Primary rat tail tendon fibroblasts were received from Columbia University. After being recovered from cryopreservation, the cells were cultured until the fourth generation in a DMEM medium supplemented with 10% FBS and 1% antibiotics (containing penicillin and streptomycin) prior to seeding. Tendon fibroblasts were seeded in both the upper and lower chambers at a density of 0.021 × 10^6^ cells/cm^2^. A total of 1.2 mL and 3 mL of the medium were added to the upper and lower chambers of the Transwell, respectively. For the ACHP experiment, a normal DMEM medium with a high glucose content (4.5 g/L) was used, whereas for the DCA experiment, DMEM with a low glucose content (1 g/L) and sodium pyruvate (110 mg/L) was used to amplify the cellular response to DCA. After adding the cell suspension, the system was shaken on a 3D shaker platform (Corning GyroTwister S1000-40, Corning Life Sciences, Durham, NC, USA) at 30 rpm for 5 min, followed by incubation at 37 °C in a humidified chamber containing 5% CO_2_ for 12 h to allow the cells to sediment and adhere to the Transwell membrane in the upper chamber and the bottom of the lower chamber.

### 2.10. Evaluation of the Biocompatibility of the Delivery System

Live/dead staining and MTT assay were used to evaluate the biocompatibility of the delivery system. Calcein-AM and EthD-1 were used to stain the live and dead cells, respectively. After 24 h of culture, cells were incubated with serum-free DMEM containing 5 mM calcein-AM and 4 μM EthD-1 at 37 °C for 30 min. After washing with PBS three times, the samples were observed under a confocal fluorescence microscope (Zeiss LSM 900). For the MTT assay, the upper and lower chambers of the Transwell system were rinsed three times with PBS and then 500 μL of serum-free DMEM containing 0.5 mg/mL MTT was added into both chambers. After incubation at 37 °C in a humidified chamber containing 5% CO_2_ for 4 h, the MTT solution was removed and 500 μL of DMSO was added to dissolve the formazan produced. The DMSO solution was then transferred to a 24-well plate and its absorbance at 570 nm was read with a microplate reader (Infinite 200, Tecan, Männedorf, Switzerland). Five technical replicates were performed for each group (N = 5).2.11. In Vitro Transwell Cell Culture Experiment for Directional Delivery of ACHP.

After tendon fibroblasts had adhered for 12 h, both the upper and lower chambers of the ACHP-treated and control group received 20 ng/mL Interleukin-1β (IL-Iβ) (R&D Systems, Minneapolis, MN, USA) for 48 h to stimulate inflammatory conditions. At the end of the culture period, the supernatants of all chambers were separately collected to evaluate inflammatory cytokine production (Interleukin 6, IL-6). The concentration of IL-6 was measured using a commercial IL-6 ELISA kit (Aviva Systems Biology, San Diego, CA, USA). The values obtained were subsequently used to calculate the total amount of IL-6 produced in each chamber by multiplying the concentration with a known total volume of the medium. The total IL-6 was normalized against the number of cells seeded in the respective chamber. RNA samples of the cells in both chambers were isolated to evaluate inflammatory-related gene expressions. 500 μL TRIzol was added to each chamber and RNA samples were isolated following the manufacturer’s protocol. Total RNA yield and sample purity were determined using a spectrophotometer (Nanodrop, Thermo Fisher, Waltham, MA, USA). 100 ng of total RNA was reverse transcribed to complementary DNA (reverse transcription kit, QIAGEN, Hilden, Germany) using a dry bath (myBlock, Benchmark Scientific, Sayreville, NJ, USA). Quantitative real-time polymerase chain reaction (PCR) was performed using TB Green and a real-time PCR System (StepOnePlus, Applied Biosystems, Waltham, MA, USA). Primers were designed using PrimerQuest (Integrated DNA Technologies, Coralville, IA, USA) and were commercially obtained from ThermoFisher Scientific. The sequences for the primers followed those published in our prior work [14]. Analysis was performed using the ΔΔCt method with *Rplp1* as the housekeeping gene.

### 2.12. In Vitro Transwell Cell Culture Experiment for Directional Delivery of DCA

After 60 h of culture, tendon fibroblasts in both the upper and lower chambers of the DCA-treated and control groups were trypsinized, snap-frozen with liquid N_2_, and pulverized by quick sonication (10 s). Acetyl-CoA concentrations in the sample extracts were determined with an acetyl-coenzyme A assay kit (Sigma-Aldrich) according to the manufacturer’s instructions. Since different numbers of cells were seeded in the upper and lower chambers of the Transwell culture system, the results were normalized against the number of cells seeded.

### 2.13. Statistical Analysis

Data are presented as mean ± standard deviation and “N” indicates the number of samples per group. One-way analysis of variance (one-way ANOVA) was performed to evaluate the generation of acetyl-CoA and the level of IL-6 production in the experiment (DCA- and ACHP-treated) and control groups; two-way analysis of variance (two-way ANOVA) was performed to evaluate the expression of inflammatory-related genes in these groups (GraphPad Prism). A significant difference was represented by a bar (** *p* < 0.01. *** *p* < 0.001, **** *p* < 0.0001).

## 3. Results and Discussion

### 3.1. Design and Characterizations of the Bi-Directional and Bi-Temporal Release System

**Figure 2.**
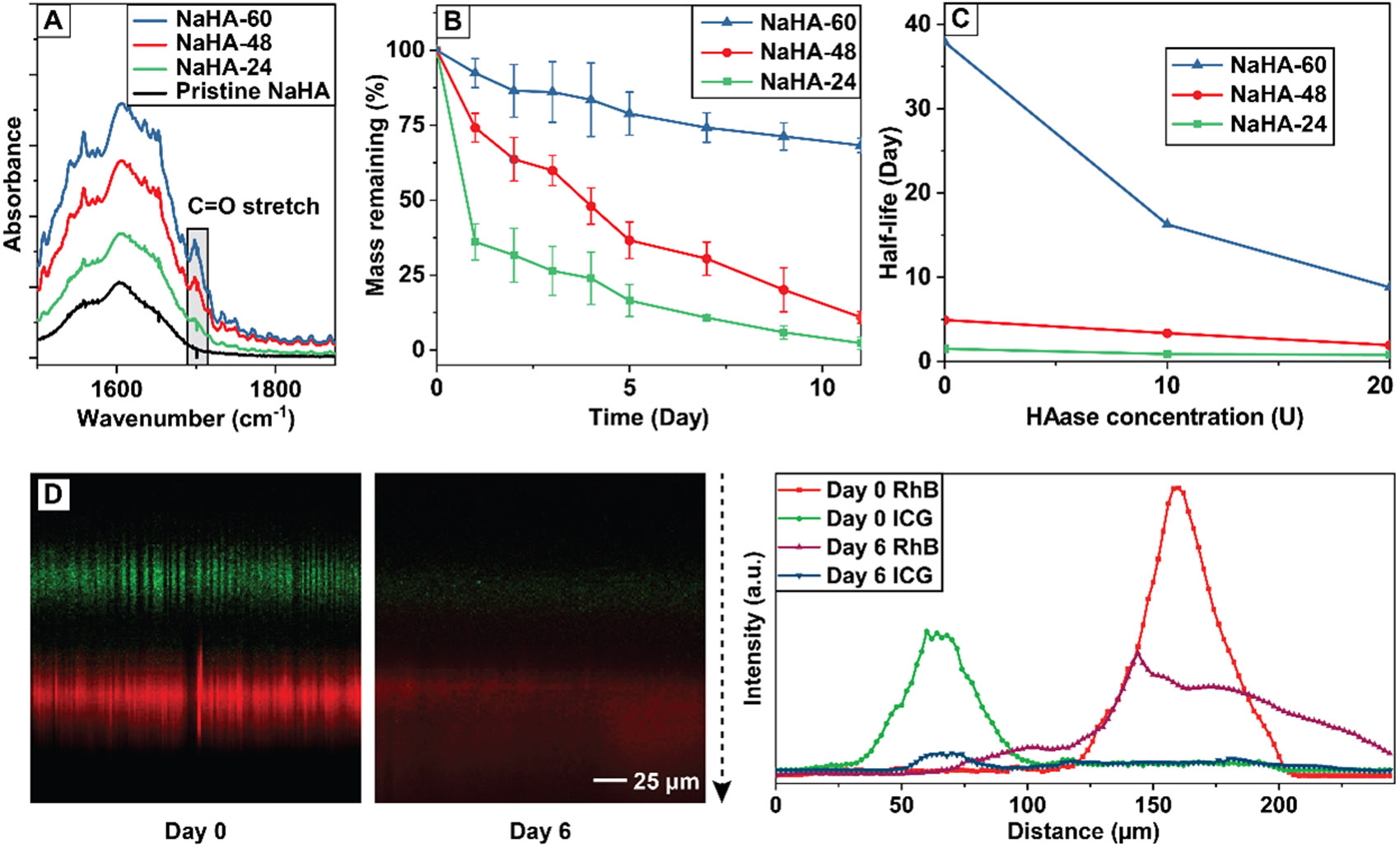
Characterizations of pristine NaHA films and those crosslinked to different degrees. (**A**) FT-IR spectra recorded from the films. (**B**) Degradation profiles of the films (N = 3 for each data point). (**C**) Half-life of the films when incubated in PBS containing different amounts of HAase (N = 3 for each data point). (**D**) Bi-directional release of fatty acid nanoparticles pre-loaded with fluorescent model compounds, ICG (green color) and RhB (red color), respectively, from the construct when incubated in enzyme-supplemented PBS at 37 °C and a quantitative analysis of the distribution of ICG and RhB following the direction of the dashed arrow (Day 0 and Day 6).

The release system was fabricated using NaHA thin films with different degrees of crosslinking. Instead of using classic crosslinking agents such as glutaraldehyde and epoxy [15,16], we chose to focus on the use of EDC for its water solubility and thus high biocompatibility of the crosslinking process. Following the protocol developed by Tomihata and Ikada [17], small pieces of NaHA thin films were immersed in an aqueous solution containing EDC and EtOH for 24, 48, and 60 h at 25 °C to achieve different degrees of crosslinking. It is worth noting that the entire process occurred under close-to-physiological pH at room temperature and did not involve any harmful organic solvents. After crosslinking, FT-IR spectra were recorded from the films, including the pristine sample, to gain a quantitative measure of the degree of crosslinking. As shown in Figures 2A and S3, the most notable difference between the spectra occurred around 1700 cm^−1^, which can be assigned to the carbonyl group (C=O stretching) associated with the new C=O bond formed during the crosslinking process. In the crosslinking process, EDC did not directly serve as a crosslinker but as an activator for the carboxylate group of the polysaccharide NaHA (Figure S4) [18]. Eventually, intermolecular ester bonds were formed between the hydroxyl and carboxyl groups of NaHA to achieve crosslinking. Compared to the initial pristine sample, the by-product of the crosslinking reaction, which was supposed to be trapped within the crosslinked polymer network, contains an additional C=O bond, contributing to the rise of the C=O stretching peak in the FT-IR spectra. Using the peak intensity around 2925 cm^−1^ for the −CH_2_ group, which did not participate in the crosslinking reaction, as a reference, the relative intensity of the C=O stretching peak increased with the duration of crosslinking (Table S1), indicating a higher degree of crosslinking.

Subsequently, we monitored the degradation of the crosslinked NaHA films in PBS supplemented with 20 units of HAase (in vivo mimicry [12]) over a period of two weeks. As shown in Figure 2B, the NaHA-60 films exhibited the slowest degradation rate, with over 60% of their original mass remaining by the end of the experimental period. In contrast, both NaHA-48 and NaHA-24 films degraded more rapidly. Specifically, NaHA-24 films lost over 50% of their original mass within the first 24 h of incubation. The cumulative release of fatty acid nanoparticles pre-loaded with a fluorescent model drug from the three types of NaHA thin films showed a trend similar to that of the degradation profile (Figure S5). Considering the presence of HAase in the in vivo environment, we monitored the release under three different conditions, with the construct being incubated in pure PBS, PBS with 10 units of HAase, and PBS with 20 units of HAase, respectively [12,13]. To quantify the release behavior, we assumed that the release process follows the first order kinetics and calculated the half-life for each type of NaHA film (Figure 2C, Table 1). As expected, increasing the concentration of HAase from 0 to 20 units significantly shortened the half-life for all three types of crosslinked HA films. At a moderate concentration of 10 units, NaHA-24 had a short half-life of around 1 day, while those of NaHA-48 and NaHA-60 were over 3 days and up to 2 weeks, respectively. These half-life values match the required time scales for inflammatory suppression in the early phase of healing and for metabolism regulation during the later proliferative and remodeling phases of healing for the in vivo repair model.

In practice, water infiltrates the looser network of the lightly-crosslinked NaHA barrier layer (NaHA-24) more rapidly when compared to the heavily-crosslinked NaHA-48 layer, leading to its disintegration within the first three days of implementation. Upon exposure to an aqueous environment, the hosting layer will swell to swiftly release the embedded fatty acid nanoparticles. While a small number of the nanoparticles may start to release their payloads (the small molecule drugs) at this stage, the major release should not occur until the nanoparticles have escaped from the crosslinked NaHA layer. When the nanoparticles are internalized by cells in vitro or come into contact with plasma albumin in body fluid in vivo, the fatty acids will be quickly dissolved or degraded, releasing the payloads [19]. Throughout this process, the most heavily-crosslinked NaHA-60 layer can prevent the inter-diffusion of nanoparticles between the two hosting layers, achieving bi-directional release.

To visualize and qualitatively analyze the release kinetics, two fluorescent compounds, ICG (green fluorescence) and RhB (red fluorescence), were selected as model compounds for ACHP and DCA, respectively. We recorded confocal fluorescence micrographs (cross-sectional view) from the construct after incubation in the enzyme-supplemented PBS at 37 °C for up to 6 days (Figure 2D, Figure S6). Due to the presence of the NaHA-60 blocking layer, the nanoparticles pre-loaded with the model compounds were released in two opposite directions with minimum inter-mixing. On day six, the signal from ICG, which represented ACHP that was supposed to be released in the early stage to combat inflammatory response, was mostly diminished. In contrast, the signal from RhB was still visible at this time point, confirming the more sustained release from this side of the construct.

In principle, NaHA films with varying degrees of crosslinking could be produced by controlling the duration of crosslinking until the maximum level is reached. As demonstrated above, the water infiltration rate is expected to be inversely correlated with the degree of NaHA crosslinking. While NaHA-24, −48, and −60 were selected based on the specific cumulative drug release requirements for the flexor tendon repair in vitro model, they can be substituted by films with other degrees of crosslinking and/or different thicknesses to achieve alternative release profiles. Furthermore, the fatty acid matrix that encapsulates the small molecule drugs could also be modified by crosslinking to offer another layer of control over the temporal release profile. The modular design grants a high level of flexibility, allowing for a broader spectrum of applications for the proposed system.

### 3.2. In Vitro Transwell Cell Culture Experiments

To demonstrate the bi-directional release capability of the delivery system in modulating cellular behavior in vitro, we adopted a Transwell culture system that allowed for the segregation of the environments. Specifically, ACHP (an IKKβ inhibitor) and DCA (a pyruvate dehydrogenase kinase 1 [PDK1] inhibitor) were delivered into the upper and lower chambers of the Transwell system. Rat tail tendon fibroblasts were seeded into both chambers, allowing us to analyze the biological effects of the directional delivery of these two effectors.

We first assessed the biocompatibility of the delivery system by incubating it with tendon fibroblasts under normal growth conditions in the Transwell system. Specifically, the delivery construct was attached to the polyethylene terephthalate (PET) membrane at the bottom of the Transwell insert using the same PVA-NaHA mixture used for the hosting layer. Through freeze-thawing cycles, physical crosslinking was established between PET, PVA, and NaHA to firmly attach the construct to the Transwell membrane throughout the cell culture experiment. Live/dead staining and MTT assay were performed for cells seeded in both the upper and lower chambers of the Transwell system. The cells showed good viability in all groups, with negligible differences between the construct-treated and control groups (Figure S7). These results indicate that the delivery system has excellent biocompatibility, which is vital to the proposed application.

Since ACHP and DCA are known to have contrasting effects on the tendon fibroblast cells, two simplified versions of the construct (Figure S8) were designed to deliver a single type of effector at a time and thereby isolate the cellular responses in one culture system. The construct, which contained either drug-loaded or plain (control group) fatty acid nanoparticles encapsulated between one barrier layer and one blocking layer of NaHA films, was attached to the Transwell insert in the same manner as described above. To better demonstrate the directionality of the delivery system, we purposely covered the entire permeable Transwell membrane with the construct to reduce the medium exchange and thereby enhance the segregation between the upper and lower chambers.

**Figure 3.**
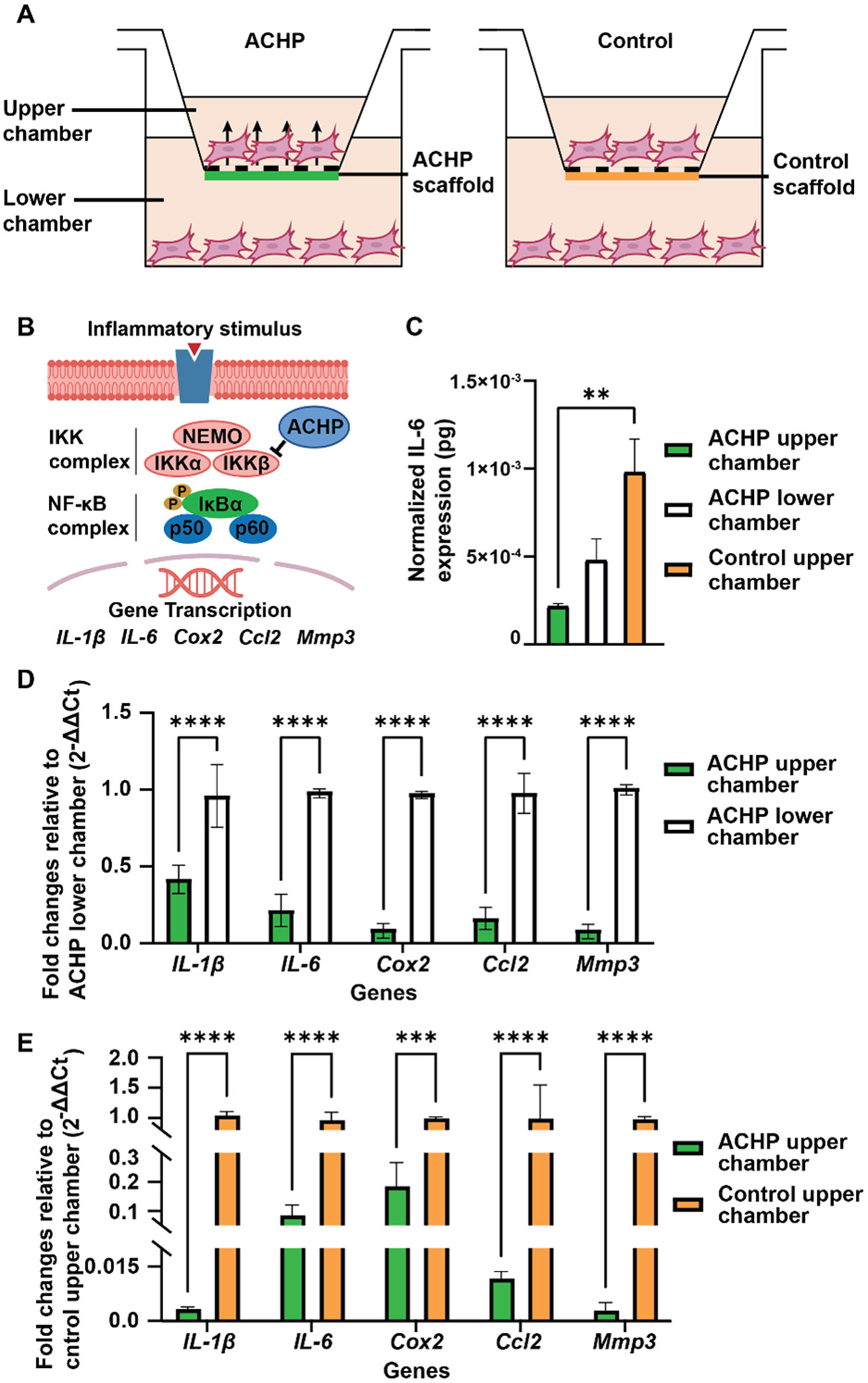
In vitro Transwell culture experiment demonstrating the directional release of ACHP. (**A**) Schematic of the experimental setup. (**B**) Schematic of the modulation of NF-κB signaling pathways by ACHP. Adapted from ref. [14]. (**C–E**) The biological effect of the directional release of ACHP as demonstrated by (**C**) reduced production of inflammatory cytokine IL-6 in the ACHP upper chamber. The value was normalized to the number of cells seeded into the upper or lower chamber of the Transwell system, respectively. (**D**) Fold changes in gene expression for the cells from the ACHP upper chamber relative to those from the ACHP lower chamber. (**E**) Fold changes in gene expression for the cells from the ACHP upper chamber relative to those from the control upper chamber. A significant difference was represented by a bar (** *p* < 0.01, *** *p* < 0.001, **** *p* < 0.0001). N = 3 for the ACHP-treated group and N = 2 for the control group.

Cell culture experiments were conducted using the ACHP-loaded constructs to demonstrate the directional release of ACHP toward the upper chamber (Figure 3A). The early inflammatory phase of healing of an intrasynovial flexor tendon is dominated by high levels of proinflammatory cytokine production and the activation of the NF-κB pathway [20]. Our previous work showed that the IKKβ inhibitor ACHP is effective in modulating inflammatory response in tendon fibroblasts in vitro and in vivo by suppressing the NF-κB pathway (Figure 3B) [14]. As a result, ACHP was designed to be released toward the tendon sheath to subdue the early inflammation and thus prevent adhesion or scar tissue formation. To model the inflammatory environment in the in vitro culture system, the tendon fibroblasts in both chambers were treated with 20 ng/mL IL-1β for 48 h after they had been seeded for 12 h. Afterward, the biological effectiveness of the directional delivery of IKKβ inhibitor ACHP was evaluated for both the ACHP delivery and control groups. Since the activation of the NF-κB pathway by IL-1β would lead to a dramatic increase in IL-6 cytokine production, we compared the concentrations of IL-6 in the ACHP upper and lower chambers with the control upper chamber. As shown in Figure 3C, the IL-6 level was significantly lower in the ACHP upper chamber (*p* < 0.01), suggesting that ACHP was predominantly released into the upper chamber, thereby effectively inhibiting the generation of IL-6. The gene expression analysis corroborated the IL-6 production result (Figure 3D,E). In the ACHP upper chamber, the expressions of NF-κB target genes (*IL-1β, IL-6*, and *Cox2*) were reduced significantly compared to the ACHP lower chamber and control upper chamber (*p* < 0.0001). In addition, the expressions of other inflammatory-related genes, such as *Ccl2*, which is involved in the recruitment of immune cells [21], and *Mmp3*, which encodes a common inflammation-promoting factor [22], were also reduced (*p* < 0.001 for comparing *Ccl2* between the ACHP and control upper chamber; *p* < 0.0001 for all other comparisons). This downregulation of inflammation-related gene expression was consistent with what was observed following systemic treatment with ACHP [14]. In contrast, there was no significant difference between the upper and lower chambers of the control group (Figure S9A,B). Collectively, it can be concluded that ACHP was successfully delivered to the upper chamber only, suppressing the NF-κB pathway and modulating inflammation.

**Figure 4.**
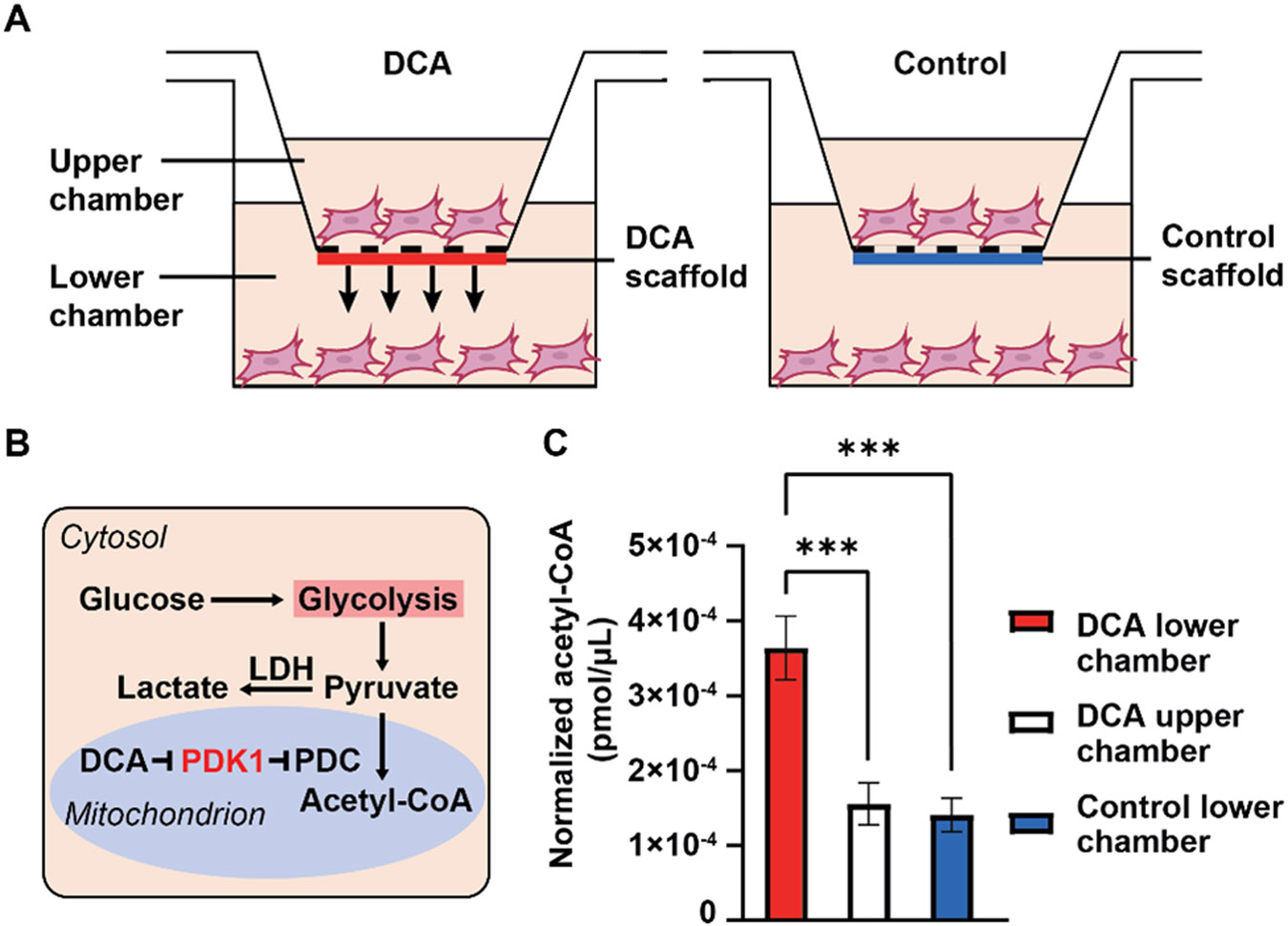
In vitro Transwell culture experiment demonstrating the directional release of DCA. (**A**) Schematic of the experimental setup. (**B**) Schematic of the primary pathways and regulation of glucose metabolism by DCA in tendon fibroblast cells. LDH, lactate dehydrogenase; PDK1, pyruvate dehydrogenase kinase 1; PDC, pyruvate dehydrogenase complex. Adapted from ref. [23]. (**C**) The biological effect of DCA as demonstrated by the increased generation of acetyl-CoA in the DCA lower chamber. The value was normalized to the number of cells seeded into the upper or lower chamber of the Transwell system, respectively. A significant difference was represented by a bar (*** *p* < 0.001). N = 4 for the DCA-treated group and N = 2 for the control group.

In the DCA cell culture experiment, the effector was designed to be released toward the lower chamber, mimicking its desired release direction toward the intrasynovial tendon in an in vivo application scenario (Figure 4A). In one of our prior studies, we reported that DCA is effective in shifting the metabolism of tendon fibroblasts from glycolysis to oxidative phosphorylation (Figure 4B) by inhibiting PDK1 [23]. Since PDK prevents pyruvate from being converted to acetyl-CoA, an inhibition of this enzyme would essentially increase the production of acetyl-CoA. To amplify the in vitro effect of DCA, we cultured the tendon fibroblasts in a pyruvate-enhanced environment. After 60 h of culture, the tendon fibroblasts were collected and pulverized. Acetyl-CoA concentrations in the sample extracts were determined. As shown in Figure 4C, within the same culture system, there was a significant increase in acetyl-CoA production in the lower chamber compared to the upper chamber as DCA was predominantly released toward the lower chamber (*p* < 0.001). Concurrently, the acetyl-CoA level in the lower chamber was also significantly higher than that in the lower chamber of the control group, where a construct loaded with plain fatty acid nanoparticles was used (*p* < 0.001). Notably, there was no significant difference between the upper chamber of the DCA release group and the lower chamber of the control group, nor between the upper and lower chambers of the control group (Figure S9C). This trend further indicates the effectiveness of the blocking layer (NaHA-60) in controlling the directionality of DCA release.

Taking together the results from the two stand-alone in vitro Transwell studies, we have demonstrated that ACHP and DCA can be effectively delivered to their targeted chambers, with minimum release toward the other side of the construct. Moreover, the biological effects of the two small molecule drugs were retained and both drugs were potent enough to induce major biological responses. This work serves as a sound basis for more comprehensive studies combining the delivery of ACHP and DCA in a single cell culture system, which would in turn serve as a strong foundation for future in vivo experiments.

## 4. Conclusions

Using NaHA, a naturally derived polymer, we have developed a bi-directional and bi-temporal controlled release system to concurrently deliver two therapeutics to address the contrasting goals of suppressing adhesion at the tendon surface and promoting tendon matrix formation within the repair site. Leveraging the distinct drug delivery capabilities of NaHA films with different degrees of crosslinking, we designed a multi-layered construct capable of releasing two payloads in opposite directions at different rates. Following validation and visualization of the delivery system, we conducted a set of Transwell cell culture experiments with tendon fibroblasts to demonstrate the system’s potential in flexor tendon repair. Two small molecule drugs, anti-adhesion ACHP and pro-matrix factor DCA, were delivered to their targeted locations to effectively modulate the cellular behaviors. The success of the in vitro study laid the groundwork for future in vivo inquiries and potential clinical translation. In principle, this delivery system can also be readily extended to deliver other combinations of therapeutics, such as cells, growth factors, and cytokines, expanding its application in tendon injury repair.

## Supplementary Material

Supporting Information

## Figures and Tables

**Table 1.** Half-life of the crosslinked NaHA films as a function of HAase concentration.

HAase Concentration (U)	NaHA-24 (Day)	NaHA-48 (Day)	NaHA-60 (Day)
0	1.5	4.9	37.9
10	0.9	3.4	16.3
20	0.8	1.9	8.8

## Data Availability

The data that support the findings of this study are available from the corresponding author, Y. Xia, upon reasonable request.
