# Supplementary material for: A Novel Bi-Directional and Bi-Temporal Delivery System for Enhancing Intrasynovial Tendon Repair
Source: Mater Interfaces. Author manuscript; Available in PMC 2025 Jul 30. (PMC12309398; doi:10.53941/mi.2024.100001)
Supplement: Supporting Information [file NIHMS2032460-supplement-Supporting_Information.pdf]

Supporting Information

A Novel Bi-Directional and Bi-Temporal Delivery System for Enhancing Intrasynovial Tendon Repair

Yidan Chen [1], Seth Kinoshita [2], Emily Yan [3], Min Hao [3], Hua Shen [4], Richard Gelberman [4], Stavros Thomopoulos [5], and Younan Xia [2,3,*]

1 School of Materials Science and Engineering, Georgia Institute of Technology, Atlanta, GA 30332, USA
2 School of Chemistry and Biochemistry, Georgia Institute of Technology, Atlanta, GA 30332, USA
3 The Wallace H. Coulter Department of Biomedical Engineering, Georgia Institute of Technology and Emory University, Atlanta, GA 30332, USA
4 Department of Orthopedic Surgery, Washington University School of Medicine, St. Louis, MO 63110, USA
5 Department of Orthopedic Surgery, Department of Biomedical Engineering, Columbia University, New York, NY 10032, USA
* Correspondence: younan.xia@bme.gatech.edu
Received: 24 September 2024; Revised: 8 October 2024; Accepted: 14 October 2024; Published: 18 October 2024

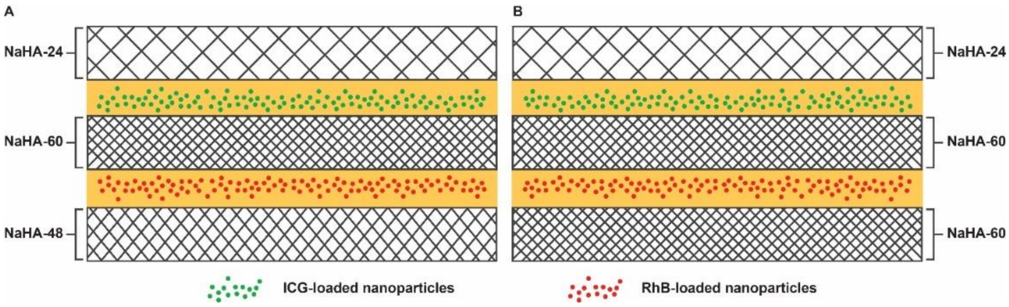

Figure S1. Schematic of the constructs used for monitoring the release of fatty acid nanoparticles from the crosslinked NaHA films. (**A**) Construct used for monitoring the release from NaHA-24 and NaHA-48. (**B**) Construct used for monitoring the release from NaHA-60.

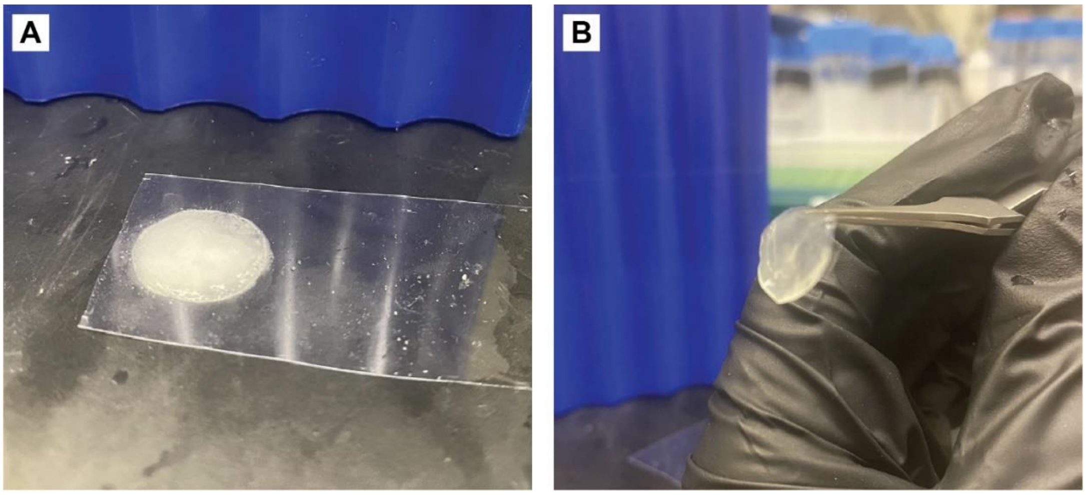

Figure S2. Digital photos of the bi-directional and bi-temporal delivery system. (**A**) A circular (10 mm in diameter) sample of the delivery system resting on a clear polytetrafluoroethylene (PTFE) substrate. (**B**) The same sample after being lifted up from the PTFE substrate with a tweezer, demonstrating its mechanical strength and pliability.

Copyright: © 2024 by the authors. This is an open access article under the terms and conditions of the Creative Commons Attribution (CC BY) license (https://creativecommons.org/licenses/by/4.0/).
Publisher's Note: Scilight stays neutral with regard to jurisdictional claims in published maps and institutional affiliations

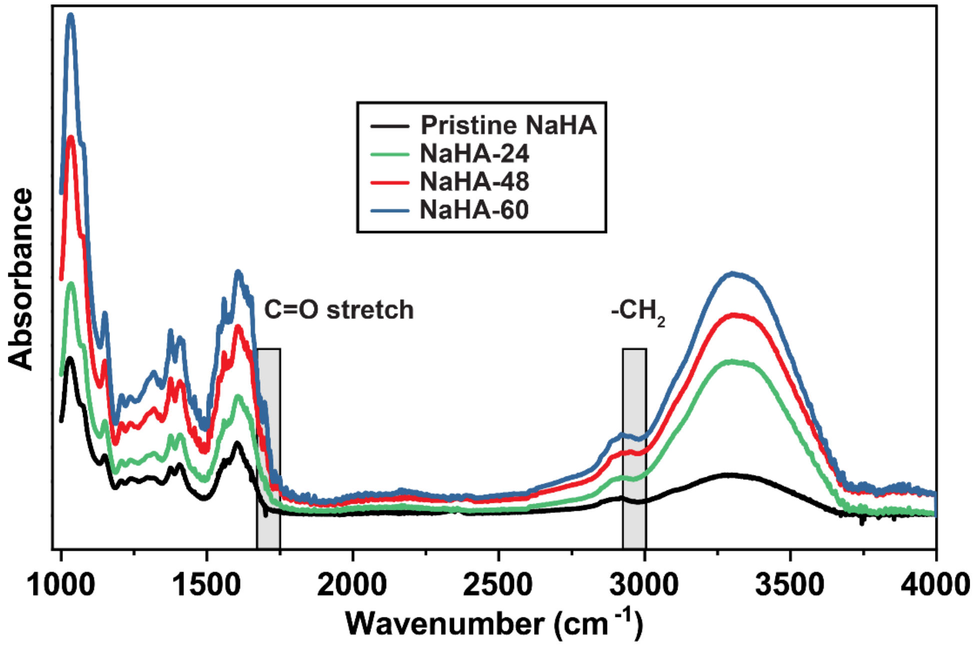

Figure S3. FT-IR spectra recorded from the NaHA films crosslinked for different periods of time. The absorbance at 2925 cm^{-1}, assigned to the -CH_2 group, is used as a reference for the relative intensity change of the C=O stretching peak [1].

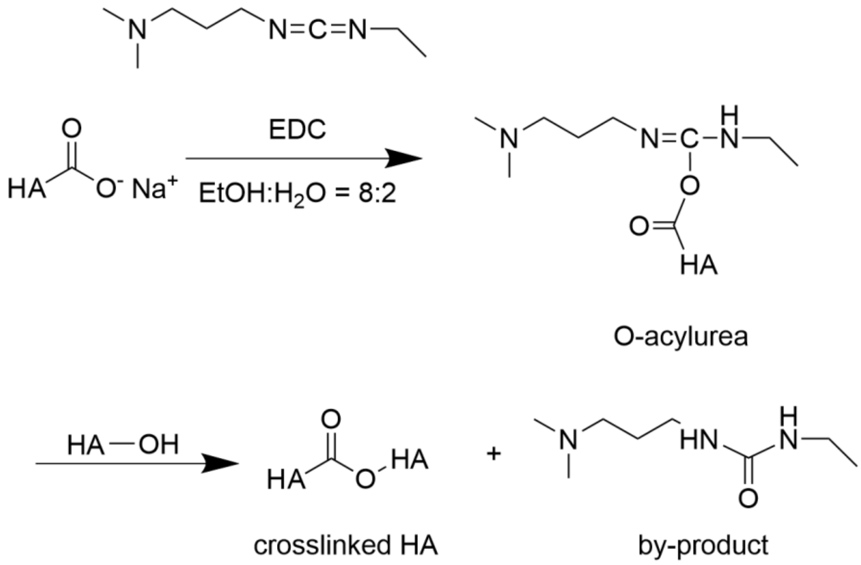

Figure S4. Schematic of the crosslinking reaction between two HA chains in the presence of EDC as an activator [2].

Table S1. Ratio between the absorbance of C=O stretching ($A_{C=O}$) and the absorbance of -CH_2 stretching ($A\text{-}_{CH2}$) for the crosslinked NaHA films.

Crosslinking Time (h)	$A_{C=O}/A\text{-}_{CH2}$
0	0.64947
24	1.28743
48	1.43047
60	1.77132

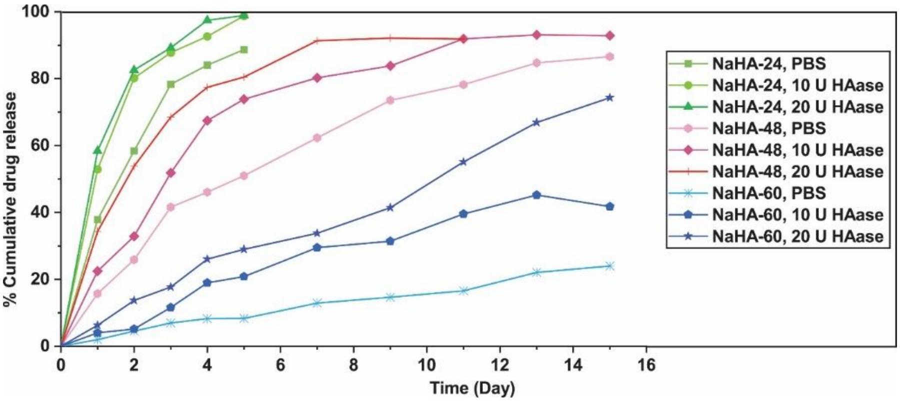

Figure S5. Cumulative release of fatty acid nanoparticles pre-loaded with fluorescent model compounds from the NaHA films with different degrees of crosslinking. The release was measured by monitoring the fluorescence intensity remaining in the NaHA films after incubation at 37 °C for different periods of time (N = 3).

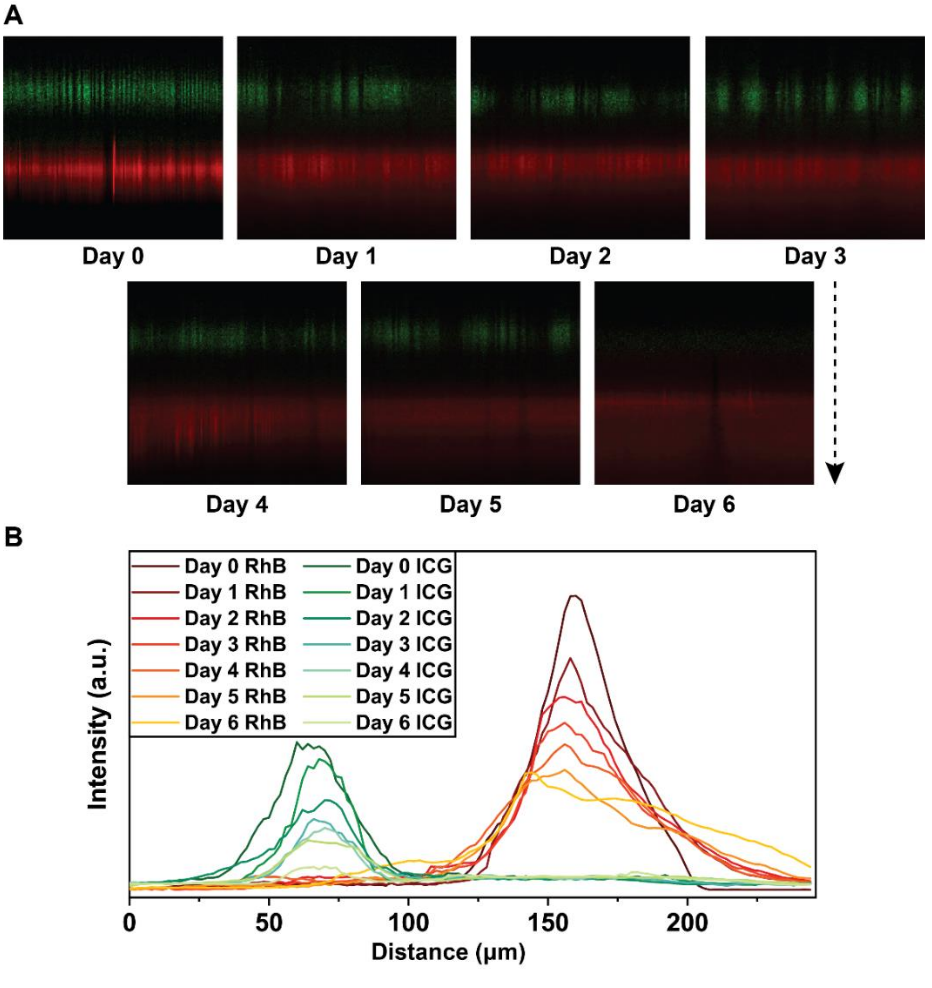

Figure S6. Bi-directional release of fatty acid nanoparticles pre-loaded with fluorescent model compounds, ICG (green color) and RhB (red color), respectively, from the construct when incubated in the enzyme-supplemented PBS at 37 °C. (**A**) Confocal fluorescence micrographs (cross-sectional view) of the construct up to six days of incubation. (**B**) A quantitative analysis of the distributions of ICG and RhB following the direction of the dashed arrow for the images in (**A**).

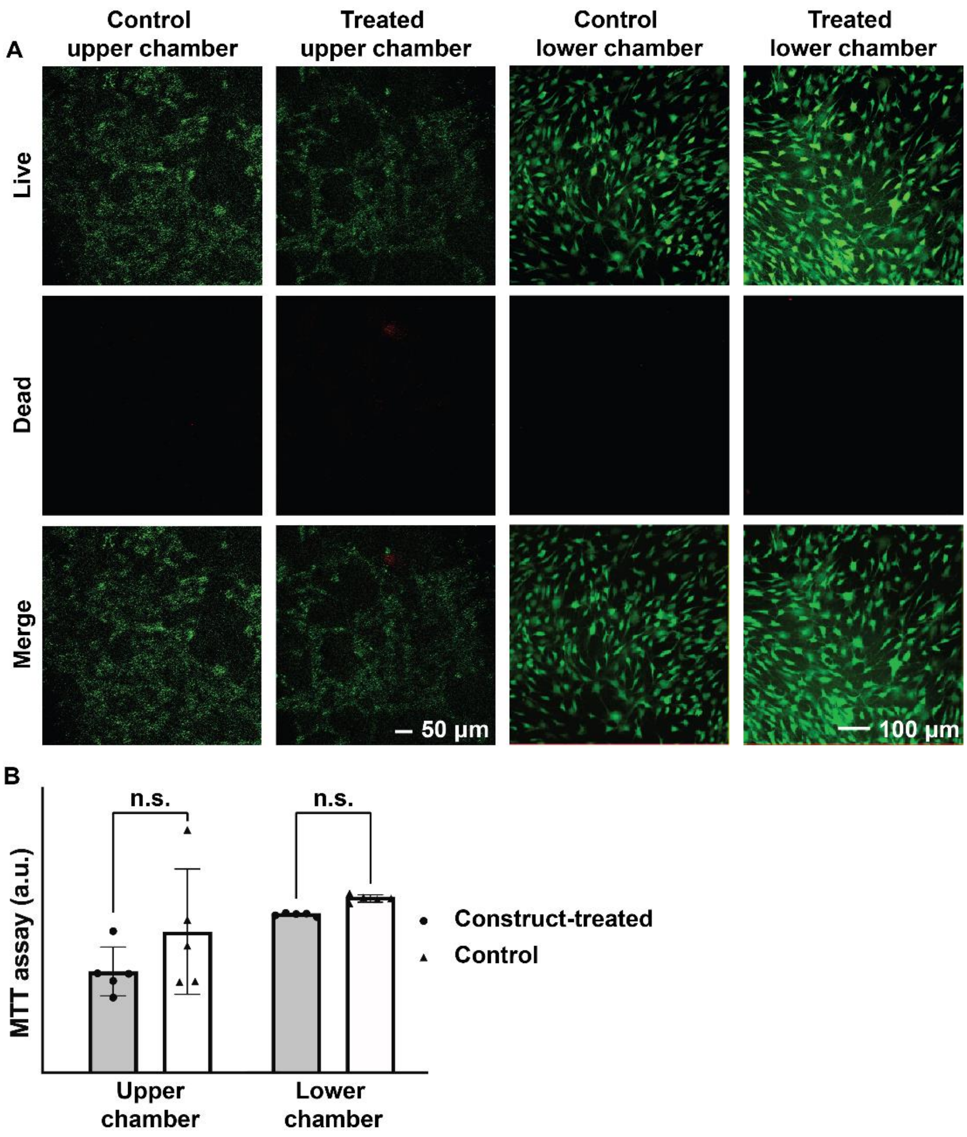

Figure S7. Biocompatibility of the delivery system. (**A**) Live/dead staining and (**B**) MTT assay result comparing the construct-treated group and the control group after 60 h of cell culture. The values were normalized against the amounts of cells seeded to the upper and lower chambers of the Transwell system, respectively. n.s., not significant. N = 5 for the MTT assay.

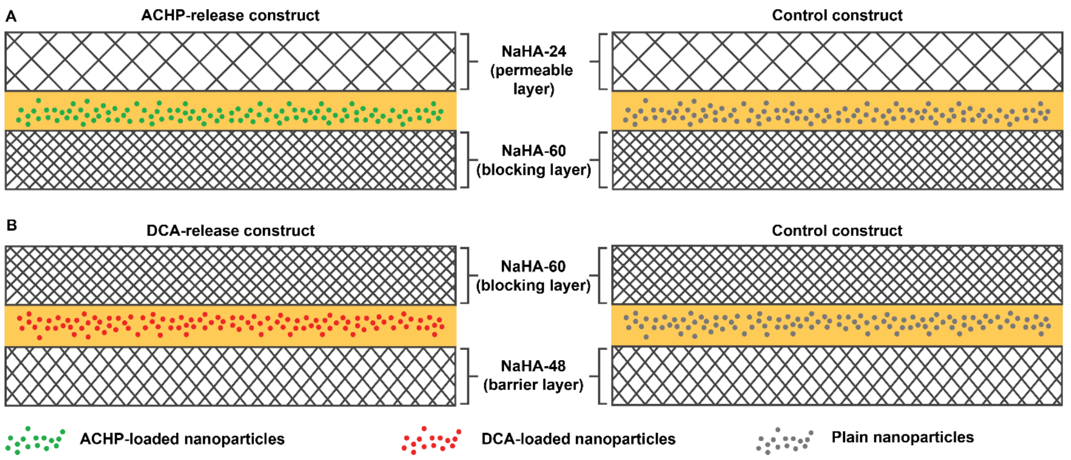

Figure S8. Schematic of the delivery systems used in the Transwell cell culture experiments. (**A**) ACHP-release construct and (**B**) DCA-release construct along with their respective control system containing plain fatty acid nanoparticles.

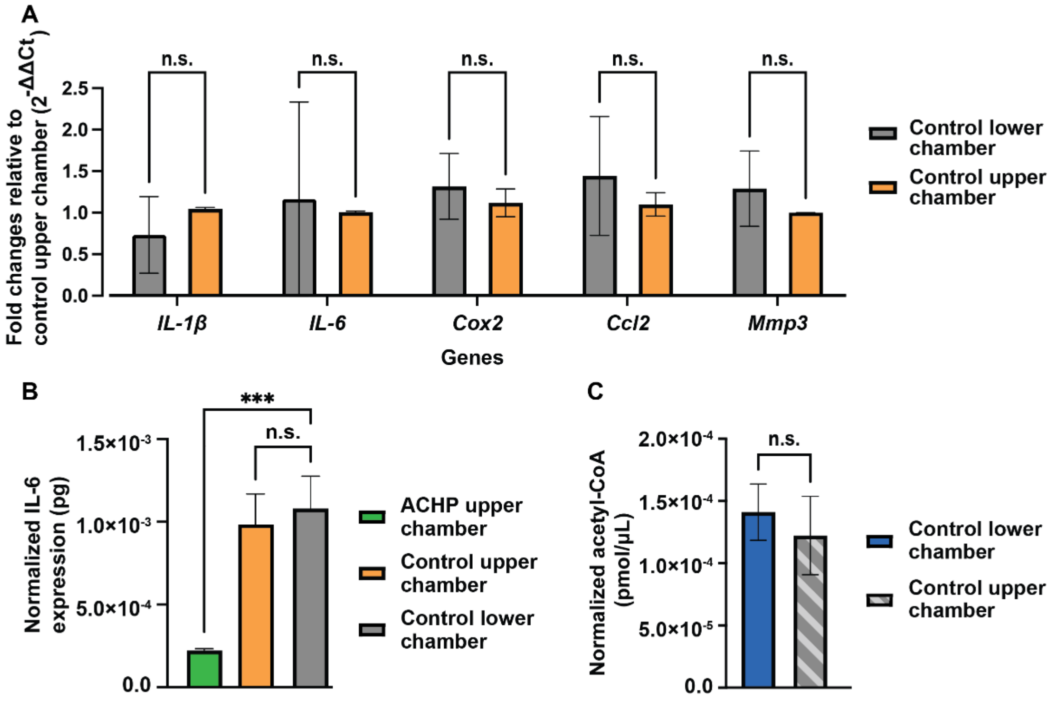

Figure S9. Comparison between the cellular response in the two chambers of the control group, where a construct loaded with plain fatty acid nanoparticles was used. (**A**) Fold changes in gene expression for the cells from the control lower chamber relative to those from the control upper chamber. (**B**) Comparison of inflammatory cytokine IL-6 production levels between the ACHP upper chamber, the control upper chamber, and the control lower chamber. The value was normalized to the number of cells seeded into the upper or lower chamber of the Transwell system, respectively. N = 3 for the ACHP-treated group and N = 2 for the control group. (**C**) Comparison of acetyl-CoA generation between the control lower chamber and the control upper chamber. The value was normalized to the number of cells seeded into the upper or lower chamber of the Transwell system, respectively. N = 4 for the DCA-treated group and N = 2 for the control group. A significant difference was represented by a bar (*** $p < 0.001$). n.s., not significant.

References

1. Tomihata, K.; Ikada, Y. Crosslinking of Hyaluronic Acid with Water-Soluble Carbodiimide. *J. Biomed. Mater. Res.* **1997**, *37*, 243–251. https://doi.org/10.1002/(sici)1097-4636(199711)37:2<243::aid-jbm14>3.0.co;2-f.
2. Lu, P.-L.; Lai, J.-Y.; Ma, D.H.-K.; Hsiue, G.-H. Carbodiimide Cross-Linked Hyaluronic Acid Hydrogels as Cell Sheet Delivery Vehicles: Characterization and Interaction with Corneal Endothelial Cells. *J. Biomater. Sci. Polym. Ed.* **2008**, *19*, 1–18. https://doi.org/10.1163/156856208783227695.
